# *N*-(2-Acetamido-2-de­oxy-β-d-gluco­pyranos­yl)-*N*-(3-azido­prop­yl)-*O*-methyl­hydroxyl­amine

**DOI:** 10.1107/S2056989016002164

**Published:** 2016-02-17

**Authors:** Stefan Munneke, Bridget L. Stocker, Mattie S. M. Timmer, Graeme J. Gainsford

**Affiliations:** a School of Chemical and Physical Sciences, Victoria University of Wellington, PO Box 600, Wellington, New Zealand; b Callaghan Innovation, PO Box 31-310, Lower Hutt 5040, New Zealand

**Keywords:** crystal structure, oxyamine glycoside, carbohydrate

## Abstract

The structure of the title compound, C_12_H_23_N_5_O_6_, solved using adequate data from a thin crystal plate, confirmed that this useful glycoconjugate was obtained in the ring-closed β-pyran­ose configuration with ^4^ *C*_1_ conformation. The mol­ecules are bound by O—H⋯O(OH) hydrogen bonds, notably in a zigzag *C*(2) chain along the short *b* (screw) axis, supplemented with an *R*_2_^2^(12) *O*—*H*⋯*O*(carbon­yl) link along the *a* axis and other *C*(2) links. The absolute configuration was not unambiguously determined but was known from the synthetic chemistry, which used natural 2-acetamido-2-de­oxy-d-glucose as the starting material.

## Abstract 2

The structure determination of an oxyamine glycoside confirmed that it was obtained in the ring-closed β-pyran­ose configuration with a ^4^ *C*_1_ conformation. The mol­ecules are bound by O—H⋯O(OH) hydrogen bonds, notably in a zigzag *C*(2) chain along the short *b* (screw) axis, supplemented with an 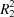
(12) *O*—*H*⋯*O*(carbon­yl) link along the *a* axis and other *C*(2) links.

## Chemical context   

Oxyamine glycosides, such as the title compound, can be utilised for the synthesis of a wide variety of complex glycoconjugates (Kwase *et al.*,2014 ▸; Munneke *et al.*, 2015 ▸; Wang *et al.*, 2013 ▸). In particular, the use of an oxyamine bifunctional linker allows for the conjugation of carbohydrates to a substrate of choice, such as proteins, fluoro­phores and biotin. The crystal structure analysis confirmed that the glycoconjugate was obtained in the ring-closed β-pyran­ose configuration.
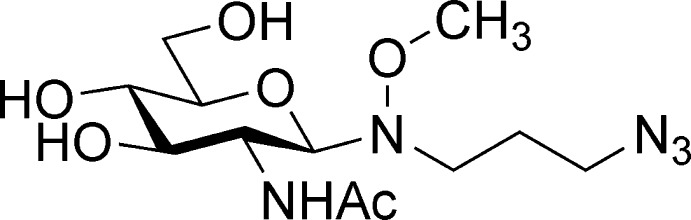


## Structural commentary   

**Figure 1:**
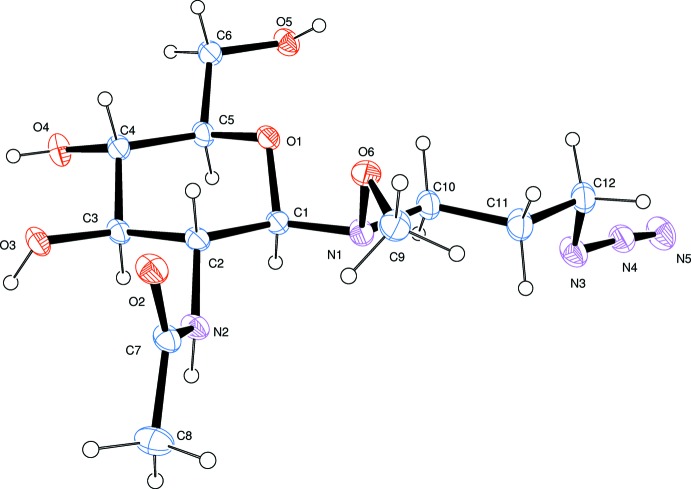
View of the title mol­ecule, drawn with 25% probability displacement ellipsoids.

The title compound crystallizes with one independent mol­ecule in the asymmetric unit (Fig. 1 ▸) in the C1(*R*), C2(*R*), C3(*R*), C4(*S*), C5(*R*) configuration. The absolute configuration was not ambiguously determined but was known from the synthetic chemistry.

## Supra­molecular features   

**Figure 2:**
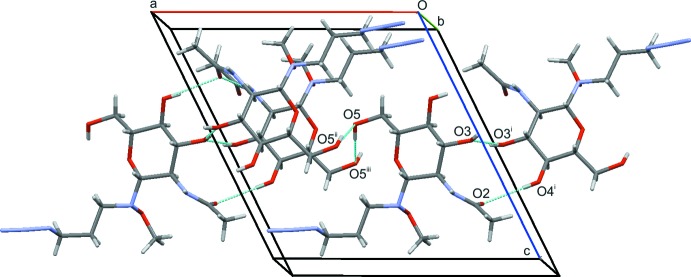
The unit-cell contents viewed along approximately the *b* axis. Some inter­molecular binding contacts are shown as blue dotted lines. [Symmetry codes: (i) −*x*, *y* + 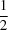, 1 − *z*; (ii) 1 − *x*, *y* − 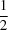, 1 − *z*; (iii) 1 − *x*, *y* + 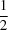, 1 − *z*.]

The mol­ecules are bound together with a comprehensive net of O—H⋯O(alcohol) hydrogen bonds, as well as one N—H⋯O(carbon­yl) and one O—H⋯O(carbon­yl) hydrogen bond (Table 1 ▸). The basic inter­actions are chain *C*(2) and *C*(8) types which combine to form a larger chain and rings *e.g. R*_2_^2^(12), as shown on the right of Fig. 2 ▸.

## Database survey   

**Figure 3:**
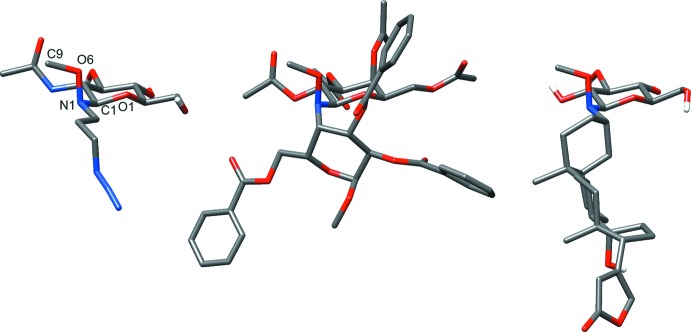
The O6—N1—C1—O1 and C9—O6—N1—C1 torsion angles of *N*-glycosyl­oxyamines: left – title compound; middle – *N*-β-gluco­pyran­osyl­oxy­amine (Langenhan *et al.*, 2005 ▸); right – *N*-β-galacto­pyran­osyl­oxy­amine (Renaudet & Dumy, 2002 ▸).

The Cambridge Structural Database (CSD, Version 5.36, update 3; Groom & Allen, 2014 ▸) was searched for *N*-alkyl-*N*-(tetra­hydro-2*H*-pyran-2-yl)oxyamines, and two structures were found, both of which are *N*-β-glycosyl­oxyamines, *viz*. an *N*-β-gluco­pyran­osyloxyamine (Langenhan *et al.*, 2005 ▸) and an *N*-β-galacto­pyran­osyloxyamine (Renaudet & Dumy, 2002 ▸). Inter­estingly, all three structures have a similar conformation around the anomeric linkage, with O6—N1—C1—O1 and C9—O6—N1—C1 torsion angles of 62.9 (8) and 115.6 (2) for the glu­cosyl­oxyamine derivative, 50.8 (1) and 126.3 (8) for the galactosyl­oxyamine and 64.2 (4) and 127.1 (3) for the title compound (Fig. 3 ▸). A configuration that allows both the meth­oxy group to adopt a pseudoaxial orientation, and positions the nitro­gen for optimal overlap between the nitro­gen lone pair and the C1—O1 σ* (n → σ* inter­action).

## Synthesis and crystallization   

*N*-(2-Acetamido-2-de­oxy-β-d-gluco­pyranos­yl)-*N*-(3-azidoprop­yl)-*O*-methyl­hydroxyl­amine was prepared as described in Munneke *et al.* (2015 ▸) from 3-azido-1-meth­oxy­amino­propane and commercially available *N*-acetyl­glucosa­mine. The title compound was recrystallized from freshly distilled MeOH–Et_2_O (1:8 *v*/*v*).

## Refinement   

Crystal data, data collection and structure refinement details are summarized in Table 2 ▸. All methyl H atoms were constrained to an ideal geometry (C—H = 0.98 Å) with *U*_iso_(H) = 1.5*U*_eq_(C), but were allowed to rotate freely about the adjacent C—C bond. All other O,C-bound H atoms were placed in geometrically idealized positions and constrained to ride on their parent atoms with C—H distances of 0.99 (methyl­ene) and 1.0 (tertiary) Å, O—H = 0.84 Å and with *U*_iso_(H) = 1.2*U*_eq_(C,O). The nitro­gen H atom was located in a difference Fourier map and refined with *U*_iso_(H) = 1.2*U*_eq_(N).

## Supplementary Material

Crystal structure: contains datablock(s) global, I. DOI: 10.1107/S2056989016002164/lh5798sup1.cif

Structure factors: contains datablock(s) I. DOI: 10.1107/S2056989016002164/lh5798Isup2.hkl

CCDC reference: 1451795

Additional supporting information:  crystallographic information; 3D view; checkCIF report

## Figures and Tables

**Table 1:** Hydrogen-bond geometry (Å, °)

*D*—H⋯*A*	*D*—H	H⋯*A*	*D*⋯*A*	*D*—H⋯*A*
O3—H3*O*⋯O3^i^	0.84	1.79	2.616 (5)	167
O4—H4*O*⋯O2^i^	0.80 (7)	2.23 (7)	3.027 (4)	170 (8)
O5—H5*O*⋯O5^ii^	0.90 (8)	1.98 (8)	2.855 (4)	167 (7)
N2—H2*N*⋯O2^iii^	0.93 (7)	2.08 (6)	2.961 (5)	158 (5)

Symmetry codes: (i) 
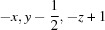
; (ii) 
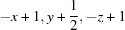
; (iii) 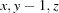.

**Table 2:** Experimental details

Crystal data	
Chemical formula	C_12_H_23_N_5_O_6_
*M*_r_	333.35
Crystal system, space group	Monoclinic, *P*2_1_
Temperature (K)	120
*a*, *b*, *c* (Å)	13.5605 (18), 4.7386 (3), 14.140 (2)
β (°)	118.181 (19)
*V* (Å^3^)	800.9 (2)
*Z*	2
Radiation type	Cu *K*α
μ (mm^−1^)	0.95
Crystal size (mm)	0.57 × 0.14 × 0.02

Data collection	
Diffractometer	Agilent SuperNova Dual Source diffractometer with an Atlas detector
Absorption correction	Gaussian (*CrysAlis PRO*; Agilent, 2014 ▸)
*T*_min_, *T*_max_	0.792, 0.985
No. of measured, independent and observed [*I* > 2σ(*I*)] reflections	6061, 2355, 2073
*R*_int_	0.053
(sin θ/λ)_max_ (Å^−1^)	0.595

Refinement	
*R*[*F* ^2^ > 2σ(*F* ^2^)], *wR*(*F* ^2^), *S*	0.059, 0.165, 1.03
No. of reflections	2355
No. of parameters	221
No. of restraints	1
H-atom treatment	H atoms treated by a mixture of independent and constrained refinement
Δρ_max_, Δρ_min_ (e Å^−3^)	0.39, −0.32
Absolute structure	Flack *x* determined using 619 quotients [(*I* ^+^)−(*I* ^−^)]/[(*I* ^+^)+(*I* ^−^)] (Parsons & Flack, 2004 ▸)
Absolute structure parameter	0.2 (4)

Computer programs: *CrysAlis PRO* (Agilent, 2014 ▸), *SHELXS97* (Sheldrick, 2008 ▸), *SHELXL2012* and *SHELXL2014* (Sheldrick, 2015 ▸), *ORTEP-3 for Windows* (Farrugia, 2012 ▸), *Mercury* (Macrae *et al.*, 2008 ▸) and *PLATON* (Spek, 2009 ▸).

## supplementary crystallographic information

### Crystal data

**Table:**

C_12_H_23_N_5_O_6_	*F*(000) = 356
*M**_r_* = 333.35	*D*_x_ = 1.382 Mg m^−^^3^
Monoclinic, *P*2_1_	Cu *K*α radiation, λ = 1.54184 Å
*a* = 13.5605 (18) Å	Cell parameters from 2327 reflections
*b* = 4.7386 (3) Å	θ = 3.7–75.3°
*c* = 14.140 (2) Å	µ = 0.95 mm^−^^1^
β = 118.181 (19)°	*T* = 120 K
*V* = 800.9 (2) Å^3^	Needle, colourless
*Z* = 2	0.57 × 0.14 × 0.02 mm

### Data collection

**Table:**

Agilent SuperNova Dual Source diffractometer with an Atlas detector	2355 independent reflections
Radiation source: sealed X-ray tube, SuperNova (Cu) X-ray Source	2073 reflections with *I* > 2σ(*I*)
Mirror monochromator	*R*_int_ = 0.053
Detector resolution: 10.6501 pixels mm^-1^	θ_max_ = 66.6°, θ_min_ = 3.6°
ω scans	*h* = −16→16
Absorption correction: gaussian (*CrysAlis PRO*; Agilent, 2014)	*k* = −5→5
*T*_min_ = 0.792, *T*_max_ = 0.985	*l* = −17→16
6061 measured reflections	

### Refinement

**Table:**

Refinement on *F*^2^	Hydrogen site location: mixed
Least-squares matrix: full	H atoms treated by a mixture of independent and constrained refinement
*R*[*F*^2^ > 2σ(*F*^2^)] = 0.059	*w* = 1/[σ^2^(*F*_o_^2^) + (0.1194*P*)^2^] where *P* = (*F*_o_^2^ + 2*F*_c_^2^)/3
*wR*(*F*^2^) = 0.165	(Δ/σ)_max_ < 0.001
*S* = 1.03	Δρ_max_ = 0.39 e Å^−^^3^
2355 reflections	Δρ_min_ = −0.31 e Å^−^^3^
221 parameters	Absolute structure: Flack *x* determined using 619 quotients [(*I*^+^)-(*I*^-^)]/[(*I*^+^)+(*I*^-^)] (Parsons & Flack, 2004)
1 restraint	Absolute structure parameter: 0.2 (4)

### Special details

**Table:**

Geometry. All e.s.d.'s (except the e.s.d. in the dihedral angle between two l.s. planes) are estimated using the full covariance matrix. The cell e.s.d.'s are taken into account individually in the estimation of e.s.d.'s in distances, angles and torsion angles; correlations between e.s.d.'s in cell parameters are only used when they are defined by crystal symmetry. An approximate (isotropic) treatment of cell e.s.d.'s is used for estimating e.s.d.'s involving l.s. planes.

### Fractional atomic coordinates and isotropic or equivalent isotropic displacement parameters (Å^2^)

**Table:**

	*x*	*y*	*z*	*U*_iso_*/*U*_eq_	
O1	0.3682 (2)	0.2828 (6)	0.5792 (2)	0.0314 (7)	
O2	0.1361 (3)	0.5357 (7)	0.7397 (3)	0.0378 (8)	
O3	0.0442 (2)	0.1637 (7)	0.5080 (3)	0.0368 (8)	
H3O	0.007 (4)	0.017 (14)	0.498 (6)	0.055*	
O4	0.1155 (3)	0.0380 (8)	0.3491 (3)	0.0426 (8)	
H4O	0.050 (6)	0.058 (17)	0.328 (5)	0.064*	
O5	0.4546 (3)	0.2456 (8)	0.4382 (3)	0.0401 (8)	
H5O	0.486 (6)	0.385 (16)	0.486 (6)	0.060*	
O6	0.4312 (3)	0.5384 (7)	0.7784 (3)	0.0369 (7)	
N1	0.4434 (3)	0.2390 (8)	0.7677 (3)	0.0329 (8)	
N2	0.2088 (3)	0.1158 (8)	0.7250 (3)	0.0326 (8)	
H2N	0.205 (4)	−0.077 (14)	0.732 (4)	0.039*	
N3	0.7789 (3)	−0.1042 (10)	0.8833 (4)	0.0480 (11)	
N4	0.8722 (3)	−0.1684 (10)	0.8948 (4)	0.0451 (10)	
N5	0.9545 (4)	−0.2571 (12)	0.9039 (4)	0.0566 (12)	
C1	0.3557 (3)	0.1498 (10)	0.6645 (4)	0.0308 (9)	
H1	0.3616	−0.0591	0.6585	0.037*	
C2	0.2369 (3)	0.2191 (9)	0.6435 (3)	0.0304 (9)	
H2	0.2276	0.4289	0.6387	0.036*	
C3	0.1552 (3)	0.0914 (9)	0.5340 (4)	0.0318 (9)	
H3	0.1626	−0.1187	0.5392	0.038*	
C4	0.1797 (3)	0.1921 (9)	0.4452 (4)	0.0320 (9)	
H4	0.1613	0.3974	0.4317	0.038*	
C5	0.3030 (3)	0.1481 (10)	0.4789 (4)	0.0338 (10)	
H5	0.3197	−0.0587	0.4869	0.041*	
C6	0.3382 (4)	0.2715 (11)	0.4006 (4)	0.0349 (9)	
H6A	0.2977	0.1734	0.3308	0.042*	
H6B	0.3172	0.4735	0.3890	0.042*	
C7	0.1536 (3)	0.2815 (10)	0.7623 (4)	0.0324 (9)	
C8	0.1154 (4)	0.1435 (11)	0.8351 (4)	0.0436 (11)	
H8A	0.1627	0.2070	0.9090	0.065*	
H8B	0.1212	−0.0619	0.8313	0.065*	
H8C	0.0375	0.1955	0.8123	0.065*	
C9	0.4282 (4)	0.5914 (10)	0.8765 (4)	0.0395 (11)	
H9A	0.4977	0.5240	0.9370	0.059*	
H9B	0.3644	0.4919	0.8757	0.059*	
H9C	0.4203	0.7945	0.8842	0.059*	
C10	0.5538 (3)	0.1952 (11)	0.7742 (4)	0.0389 (11)	
H10A	0.5617	0.3227	0.7227	0.047*	
H10B	0.5589	−0.0014	0.7532	0.047*	
C11	0.6489 (4)	0.2502 (12)	0.8862 (4)	0.0443 (11)	
H11A	0.6441	0.4474	0.9068	0.053*	
H11B	0.6405	0.1240	0.9378	0.053*	
C12	0.7623 (4)	0.2026 (12)	0.8934 (4)	0.0451 (12)	
H12A	0.7675	0.3074	0.8353	0.054*	
H12B	0.8216	0.2735	0.9630	0.054*	

### Atomic displacement parameters (Å^2^)

**Table:**

	*U*^11^	*U*^22^	*U*^33^	*U*^12^	*U*^13^	*U*^23^
O1	0.0257 (13)	0.0269 (16)	0.0409 (16)	−0.0022 (12)	0.0150 (12)	0.0014 (13)
O2	0.0370 (16)	0.0236 (16)	0.056 (2)	0.0021 (13)	0.0245 (15)	0.0010 (15)
O3	0.0235 (13)	0.0265 (16)	0.060 (2)	−0.0025 (12)	0.0190 (14)	−0.0003 (15)
O4	0.0270 (15)	0.047 (2)	0.0476 (19)	−0.0046 (16)	0.0129 (14)	−0.0117 (17)
O5	0.0322 (15)	0.0397 (19)	0.0533 (19)	−0.0004 (14)	0.0243 (14)	−0.0017 (17)
O6	0.0412 (17)	0.0222 (15)	0.0421 (17)	−0.0018 (14)	0.0152 (14)	0.0019 (14)
N1	0.0277 (17)	0.0250 (19)	0.042 (2)	0.0011 (14)	0.0130 (15)	0.0004 (15)
N2	0.0313 (17)	0.0212 (18)	0.049 (2)	0.0013 (14)	0.0223 (16)	0.0030 (15)
N3	0.033 (2)	0.042 (2)	0.066 (3)	−0.0007 (19)	0.021 (2)	0.001 (2)
N4	0.038 (2)	0.042 (2)	0.051 (2)	−0.0025 (18)	0.0174 (19)	0.0016 (19)
N5	0.036 (2)	0.055 (3)	0.079 (3)	0.003 (2)	0.027 (2)	−0.002 (3)
C1	0.0266 (19)	0.027 (2)	0.037 (2)	0.0008 (17)	0.0136 (17)	0.0035 (17)
C2	0.0266 (19)	0.022 (2)	0.045 (2)	0.0031 (16)	0.0187 (18)	0.0052 (18)
C3	0.0256 (19)	0.021 (2)	0.047 (2)	0.0000 (16)	0.0160 (18)	0.0006 (18)
C4	0.027 (2)	0.026 (2)	0.041 (2)	0.0014 (17)	0.0143 (18)	0.0008 (19)
C5	0.0249 (19)	0.033 (2)	0.042 (2)	0.0018 (18)	0.0146 (17)	0.0022 (19)
C6	0.033 (2)	0.034 (2)	0.039 (2)	−0.002 (2)	0.0172 (18)	0.000 (2)
C7	0.0297 (19)	0.022 (2)	0.045 (2)	−0.0016 (17)	0.0173 (18)	−0.0012 (18)
C8	0.056 (3)	0.028 (2)	0.061 (3)	0.000 (2)	0.039 (2)	0.003 (2)
C9	0.042 (2)	0.030 (2)	0.041 (2)	−0.0021 (19)	0.016 (2)	−0.004 (2)
C10	0.027 (2)	0.040 (3)	0.044 (3)	0.0000 (19)	0.0112 (19)	0.001 (2)
C11	0.034 (2)	0.044 (3)	0.046 (3)	0.001 (2)	0.012 (2)	−0.001 (2)
C12	0.033 (2)	0.040 (3)	0.048 (3)	−0.002 (2)	0.007 (2)	0.000 (2)

### Geometric parameters (Å, º)

**Table:**

O1—C5	1.420 (6)	C3—C4	1.520 (6)
O1—C1	1.441 (5)	C3—H3	1.0000
O2—C7	1.240 (6)	C4—C5	1.521 (5)
O3—C3	1.413 (5)	C4—H4	1.0000
O3—H3O	0.84 (8)	C5—C6	1.515 (6)
O4—C4	1.421 (6)	C5—H5	1.0000
O4—H4O	0.80 (7)	C6—H6A	0.9900
O5—C6	1.413 (5)	C6—H6B	0.9900
O5—H5O	0.90 (8)	C7—C8	1.502 (6)
O6—C9	1.430 (6)	C8—H8A	0.9800
O6—N1	1.445 (5)	C8—H8B	0.9800
N1—C1	1.443 (6)	C8—H8C	0.9800
N1—C10	1.470 (5)	C9—H9A	0.9800
N2—C7	1.352 (6)	C9—H9B	0.9800
N2—C2	1.459 (5)	C9—H9C	0.9800
N2—H2N	0.93 (7)	C10—C11	1.520 (6)
N3—N4	1.235 (6)	C10—H10A	0.9900
N3—C12	1.488 (7)	C10—H10B	0.9900
N4—N5	1.141 (6)	C11—C12	1.510 (7)
C1—C2	1.529 (5)	C11—H11A	0.9900
C1—H1	1.0000	C11—H11B	0.9900
C2—C3	1.539 (6)	C12—H12A	0.9900
C2—H2	1.0000	C12—H12B	0.9900
			
C5—O1—C1	112.0 (3)	C6—C5—H5	109.1
C3—O3—H3O	109.5	C4—C5—H5	109.1
C4—O4—H4O	112 (5)	O5—C6—C5	111.9 (3)
C6—O5—H5O	106 (5)	O5—C6—H6A	109.2
C9—O6—N1	109.4 (3)	C5—C6—H6A	109.2
C1—N1—O6	108.2 (3)	O5—C6—H6B	109.2
C1—N1—C10	110.6 (3)	C5—C6—H6B	109.2
O6—N1—C10	107.2 (3)	H6A—C6—H6B	107.9
C7—N2—C2	120.9 (4)	O2—C7—N2	122.5 (4)
C7—N2—H2N	118 (3)	O2—C7—C8	120.9 (4)
C2—N2—H2N	118 (3)	N2—C7—C8	116.6 (4)
N4—N3—C12	114.8 (4)	C7—C8—H8A	109.5
N5—N4—N3	172.6 (6)	C7—C8—H8B	109.5
O1—C1—N1	110.6 (3)	H8A—C8—H8B	109.5
O1—C1—C2	105.9 (3)	C7—C8—H8C	109.5
N1—C1—C2	115.1 (4)	H8A—C8—H8C	109.5
O1—C1—H1	108.4	H8B—C8—H8C	109.5
N1—C1—H1	108.4	O6—C9—H9A	109.5
C2—C1—H1	108.4	O6—C9—H9B	109.5
N2—C2—C1	114.7 (3)	H9A—C9—H9B	109.5
N2—C2—C3	109.3 (3)	O6—C9—H9C	109.5
C1—C2—C3	107.6 (3)	H9A—C9—H9C	109.5
N2—C2—H2	108.4	H9B—C9—H9C	109.5
C1—C2—H2	108.4	N1—C10—C11	112.2 (4)
C3—C2—H2	108.4	N1—C10—H10A	109.2
O3—C3—C4	109.3 (4)	C11—C10—H10A	109.2
O3—C3—C2	109.8 (3)	N1—C10—H10B	109.2
C4—C3—C2	111.9 (3)	C11—C10—H10B	109.2
O3—C3—H3	108.6	H10A—C10—H10B	107.9
C4—C3—H3	108.6	C12—C11—C10	112.3 (4)
C2—C3—H3	108.6	C12—C11—H11A	109.1
O4—C4—C3	110.8 (4)	C10—C11—H11A	109.1
O4—C4—C5	108.4 (3)	C12—C11—H11B	109.1
C3—C4—C5	109.5 (3)	C10—C11—H11B	109.1
O4—C4—H4	109.4	H11A—C11—H11B	107.9
C3—C4—H4	109.4	N3—C12—C11	109.5 (4)
C5—C4—H4	109.4	N3—C12—H12A	109.8
O1—C5—C6	107.1 (3)	C11—C12—H12A	109.8
O1—C5—C4	109.0 (3)	N3—C12—H12B	109.8
C6—C5—C4	113.4 (4)	C11—C12—H12B	109.8
O1—C5—H5	109.1	H12A—C12—H12B	108.2
			
C9—O6—N1—C1	127.1 (3)	C2—C3—C4—O4	170.7 (3)
C9—O6—N1—C10	−113.6 (4)	O3—C3—C4—C5	173.0 (4)
C5—O1—C1—N1	164.6 (3)	C2—C3—C4—C5	51.1 (5)
C5—O1—C1—C2	−70.2 (4)	C1—O1—C5—C6	−170.3 (3)
O6—N1—C1—O1	64.2 (4)	C1—O1—C5—C4	66.7 (4)
C10—N1—C1—O1	−52.9 (5)	O4—C4—C5—O1	−175.5 (3)
O6—N1—C1—C2	−55.6 (4)	C3—C4—C5—O1	−54.5 (5)
C10—N1—C1—C2	−172.8 (4)	O4—C4—C5—C6	65.3 (5)
C7—N2—C2—C1	136.0 (4)	C3—C4—C5—C6	−173.7 (4)
C7—N2—C2—C3	−103.1 (4)	O1—C5—C6—O5	55.3 (5)
O1—C1—C2—N2	−176.8 (3)	C4—C5—C6—O5	175.6 (4)
N1—C1—C2—N2	−54.4 (5)	C2—N2—C7—O2	−8.8 (7)
O1—C1—C2—C3	61.4 (4)	C2—N2—C7—C8	172.2 (4)
N1—C1—C2—C3	−176.2 (3)	C1—N1—C10—C11	−172.5 (4)
N2—C2—C3—O3	58.2 (4)	O6—N1—C10—C11	69.7 (5)
C1—C2—C3—O3	−176.7 (3)	N1—C10—C11—C12	179.5 (4)
N2—C2—C3—C4	179.7 (3)	N4—N3—C12—C11	−175.7 (4)
C1—C2—C3—C4	−55.1 (4)	C10—C11—C12—N3	−69.7 (6)
O3—C3—C4—O4	−67.5 (4)		

### Hydrogen-bond geometry (Å, º)

**Table:**

*D*—H···*A*	*D*—H	H···*A*	*D*···*A*	*D*—H···*A*
O3—H3*O*···O3^i^	0.84	1.79	2.616 (5)	167
O4—H4*O*···O2^i^	0.80 (7)	2.23 (7)	3.027 (4)	170 (8)
O5—H5*O*···O5^ii^	0.90 (8)	1.98 (8)	2.855 (4)	167 (7)
N2—H2*N*···O2^iii^	0.93 (7)	2.08 (6)	2.961 (5)	158 (5)

Symmetry codes: (i) −*x*, *y*−1/2, −*z*+1; (ii) −*x*+1, *y*+1/2, −*z*+1; (iii) *x*, *y*−1, *z*.
